# An Exposition of the Signs and Symptoms of Pregnancy; with Some Other Papers on Subjects Connected with Midwifery

**Published:** 1857-03

**Authors:** 


ART. V.—*An Exposition of the Signs and Symptoms of Pregnancy; with some other Papers on Subjects connected with Midwifery.* By W. F. MONTGOMERY, M.D., &c. &c., of Dublin. Second edition. 8vo., pp. 706. London, 1856.

THE first edition of Dr. Montgomery's treatise on the Signs of Pregnancy was issued many years since. It at once became the standard work, as it was almost the only one in any language on the subject, which position it has retained till now, not merely in Great Britain and this country, but, by its translations, throughout the continent of Europe.

Such having been the acknowledged value of the early edition, that of the volume just issued, based on a so much more extended experience, may be premised from the author's own words: "The present treatise, although only another edition of a work already published, may be, I think, with truth regarded as almost a new book, every sentence of the former having been carefully revised, and the quantity of new matter now added being more than equal in amount to the whole contents of the former edition."

Thirteen chapters are devoted by Dr. Montgomery to the signs and symptoms of pregnancy, and three others to the normal period of human gestation, the signs of delivery, the spontaneous amputation of fœtal limbs in utero, and some other pathological lesions (of especial interest legally) to which the child is liable before birth.

These several subjects, so interesting in themselves, and so important, every one of them, to the most delicate and precious of social relations, controlling often the honor and domestic peace of a family, the legitimacy of offspring, or the life of its parent, are all treated with an elegance of diction, fulness of illustration, acuteness and justice of reasoning, unparalleled in obstetrics and unsurpassed in medicine. The reader's interest can never flag, so fresh, and vigorous, and classical is our author's style; and one forgets, in the renewed charm of every page, that it, and every line, and every word has been weighed and reweighed through years of preparation; that this is of all others the book of Obstetric Law, on each of its several topics; on all points connected with pregnancy, to be everywhere received as a manual of special jurisprudence, at once announcing fact, affording argument, establishing precedent, and governing alike the juryman, advocate, and judge.

It is not merely in its legal relations that we find this work so interesting. Hardly a page but that has its hints or facts important to the general practitioner; not a chapter without especial matter for the anatomist, physiologist, or pathologist. The researches of our own countrymen on the corpus luteum and kyestein, are worthily remembered; and though in neither instance does Dr. Montgomery fully agree with these observers, he has yet done very much to render permanent the reputation of Dalton,* and the world-renowned E. K. Kane.

Our limits prevent such an analysis of the book as a whole or of any of its parts, as we should desire, while we deprecate the too general custom of merely quoting from preface or table of contents. We shall therefore confine ourselves to brief mention of a single topic, selected only because we are here compelled to dissent in some respects from Dr. Montgomery's conclusions.

On this point, the RELATIVE VALUE of the signs of pregnancy, decision is not easy—impossible, indeed, except by such elaborate and careful comparisons as are here furnished us.

Dr. Montgomery's division of all the ordinary signs of pregnancy† into presumptive, probable, and unequivocal, is both simple and convenient. The first group is very naturally made to comprise those constitutional, sympathetic, or distant symptoms which generally or frequently accompany pregnancy; suppression of the catamenia; irritability of the nervous system, as shown by body or mind; the changes undergone in character or function by the breast, stomach, salivary glands, or kidneys; the umbilical areola, and the dark abdominal line.

To the second group, are assigned the alterations, of whatever kind, evident during life in the uterus and its appendages, among which, not the least important, is the dusky hue of the vagina.

In the third class, Dr. Montgomery includes those evidences of the presence of a fœtus in utero given by palpation, external and internal, by auscultation, by actual inspection of the uterine contents when discharged, and by post-mortem examination of the organs themselves.

We need say nothing of the first and second groups of symptoms, those presumptive and probable, for all are familiar with their worthlessness when existing alone, and their frequent fallaciousness when combined. Not even from the well-developed mammary areola or the abundant presence of kyestein, ought a decided opinion to be ever given. We have seen the first in more than one case of uterine irritation unaccompanying pregnancy, and the last not uncommonly in dyspepsia, even in the male.

* Since the above was written, Dr. Dalton has very ably defended his positions with regard to the corpus luteum in the American Journal of the Medical Sciences for Jan. 1857, p. 113.

† Page 73.

Upon what sign of pregnancy, then, passing by, in this connection, the evidence given by abortion or labor, as also that from post-mortem inspection, and studying only the living mother, can we best rely as certain and reliable?

Upon three signs, says Dr. Montgomery:*

"1. Active movements of the child, unequivocally felt by another.

"2. Its presence in utero ascertained by ballottement.

"3. The pulsations of the fœtal heart."

In a previous page,† the placentary sound or uterine souffle is also spoken of as an unequivocal sign, but subsequently,‡ this opinion is very properly retracted. We ourselves have heard from vascular abdominal tumors sounds impossible in any way to be distinguished from the true murmur of pregnancy.

"If any one of these three signs," we are told, "be ascertained beyond doubt, it settles the question" of pregnancy. Is it, however, possible to ever ascertain each of them beyond all doubt?

When the fœtal pulsation can be heard, there can indeed be no doubt; but we do not think the same can be said of the others, particularly of those sensations of motion appreciable by the hand, whether they arise from causes independent or otherwise of the hand's action.

Evident movements of the patient's abdomen might be thought to leave little room for mistake, if discovered by inspection merely, much less if perceived by the observer's touch; and there are few practitioners, even of large experience, who would believe themselves liable to error on this point. Nevertheless, there are sources of such error, which, in some cases, there being no other reliable sign present, can only be guarded against by waiting patiently till the end of the normal period of pregnancy.

We say nothing of the movements of the intestines or their contents, which often simulate those of a child; nor of that new sign lately proposed by Dr. Oldham, of Guy's Hospital, as "a trustworthy characteristic," the contraction of the uterus itself under external manipulation, a sign which Dr. Montgomery has neglected even to mention, and which, after repeated trials, we are inclined to think very doubtful and uncertain. Still less would we speak of those sensations which are evident only to the patient herself, so often illusory; nor of those voluntary movements on her part, exhibited in cases of malingering, although these can at times be detected and checked only by the use of anæsthetics.

But it is to another large class of cases that we refer; to those which have deceived, as Dr. Montgomery allows, "very experienced and competent judges,"—for instance, Dewees, Ingleby, and even himself, where the movements are unintended, perhaps even unfelt, by the mother, though perfectly apparent to others. One striking and famous instance of this kind we repeatedly had an opportunity of examining in Edinburgh. The abdominal motions were here entirely involuntary and irregular, but so constant and well-marked as to have frequently deceived the shrewdest experts; and the husband, after careful auscultation, examination with the sound, and, above all, time had demonstrated that his wife's abdomen certainly contained no child, not even an extra uterine, still avowed that he could not disbelieve his own eyes, and that the motion must then be from a beast. Sometimes this anomaly may be explained by irregular action of the recti or other abdominal muscles, it being almost always accompanied by the usual signs of so-called spurious pregnancy, and its subjects generally hysterical; but in other cases it cannot as yet be accounted for. The very fact, however, of its occasional existence renders it necessary for all abdominal motions to be cut off from the group of absolutely certain and unequivocal signs. We doubt, indeed, if Dr. Montgomery would have so included them, had he noticed how completely he himself was begging this question when he declared, as one of his three "certain and unequivocal symptoms," "*active movements of the child, unequivocally felt by another.*"* We are compelled, therefore, to consider the first of the three signs, so far as its legal value is concerned, strongly so though it may be, as but presumptive.

Can ballottement, the second sign under consideration, be also simulated? We consider that it sometimes can. Dr. Montgomery, on the other hand, declares it "proof positive of a fœtus in utero."† He himself, however, gives cases in which Depaul and Cazeaux were deceived by simple uterine displacements, which gave to the finger "exactly the sensation" of a fœtus. Such cases, we are satisfied, are not uncommon, and are only unnoticed because unsearched for. One would hardly suppose a stone in the bladder to be thus mistaken, unless in a case of that strange, though at times perfectly successful experiment of the present day, where, to cure vesico-vaginal or vesico-uterine fistula, the os and cervix uteri are stitched into the cavity of the bladder; but Dr. Montgomery has known the pulsation of an artery through the vaginal wall pronounce the ascent and return of a fœtal head. We ourselves have felt a similar sensation given by the rebound of an intra-uterine polypus; in some cases by a small and pediculated extramural fibrous tumor, while, on the other hand, the return wave of fluctuation in menses retained, or in hydrometra, requires no ordinary touch to determine. Ballottement through the external abdominal wall must always be an extremely uncertain sign, as much so indeed as the simple percussions, in test of pregnancy, we have heard by Piorry's most unreliable hand. Ballottement per vaginam is of course much less decep-

* The italics are our own. † Page 200.

tive, but still we think hardly to be called unequivocal. We have waived all mention of its inefficacy in placenta prævia, in most cases before the sixth month, and in every case after it where there happens to be any malpresentation of the child; and were we to confine our definition of pregnancy to its closest limits, as within only its longest possible normal term, though we ignore utterly that cruel and absurd point of law "quick with child," the possibility of mistake would seem yet greater; even if all cases of extra-uterine gestation were set aside. Children are sometimes retained, as in the case of a "secondary fœtus," and some forms of arrest of development or of destructive degeneration, months and even years in the uterus after the usual days of pregnancy, and even of the very capacity for impregnation, have ended, and it is not always that the uterus in these cases is found entirely empty of fluid.

We well know that the successive stages of pregnancy at which the various signs are distinguishable, justly give to each and all of them various degrees of relative importance, but we are now discussing the actual value of individual signs by themselves, so long as they are present, no matter when.

How is it, lastly, with the third of Dr. Montgomery's signs,—the pulsation of a fœtal heart?

To the consideration of this point Dr. Montgomery has devoted many pages, candidly confessing its every advantage, skilfully refuting every objection as yet urged against it, pointing out everything with which it could possibly chance to be confounded. By so doing he has greatly strengthened the opinion to which we were bred, and which our experience has always confirmed, that the fœtal pulsation is, when heard, not only an always certain sign of pregnancy, but in every case the *only* certain one.

But we strongly dissent from his closing remarks on the subject, intended as these evidently are, to express in a word his opinion.* "Having thus fully considered the several phenomena connected with pregnancy discoverable by auscultation, and with some years' additional experience, I feel bound to repeat the opinion which I formerly gave with reference to its application for the purpose of ascertaining the existence of pregnancy; and must again say, that without meaning to depreciate its great and acknowledged value as an *occasional* means of doing so, I am still of opinion that for those who are thoroughly conversant with the proper methods of examining doubtful cases, it will seldom be found necessary, if all the other more ordinary modes have been adopted with sufficient care; that if we inquire carefully and minutely into the history of our case, and know how to make good use of our hands and eyes, we will not often require the assistance of our ears."

* We quote from pages 237 and 238.

To our mind, the use of the ear in the diagnosis of pregnancy, of course mediately by the stethoscope, is much more satisfactory than sight or touch, because, as we have seen, where there is any positive evidence it gives it, and is therefore more certain, and infinitely more decent, besides being in almost every respect and instance much easier to propose and to practise for both patient and physician.

We consider this sign, the fœtal pulse, carefully distinguished from a quickened beat of the mother's heart, and from that of the listener himself, the last a frequent source of error, but one that Dr. Montgomery has entirely neglected to mention,—the only one on which a decided opinion ought ever to be formed that may be called for by a court of law, and the first one that should be sought in general practice. If it be obtained, much disagreeable questioning and all manipulation will, at once, be rendered unnecessary, and in many cases, therefore, unjustifiable.*

With thanks, for the gratification it has afforded, we have closed his book. It has freshened our recollections of the cheery, genial face, the thoughtful mind, and, better than all, the kind heart of whose existence we have had abundant proof.

* As we write, we learn Dr. Montgomery's resignation of the chair he has long and so worthily held; but, we are sure, he retires from its active duties with no intention of resting from the patient and laborious researches that have gained him such fame.

